# Diet in Disease

**Published:** 1892-10-01

**Authors:** Ernest Hart

**Affiliations:** Bachelier-es-Sciences-es-Lettres (restreint), Medical Student of the Faculty of Medicine of Paris, and of the London School of Medicine for Women


### DIET IN DISEASE.

By Mrs. Ernest Hart,* Bachelier-es-Sciences-es-Lettres (restreint), Medical Student of the Faculty of Medicine of Paris, and of the London School of Medicine for Women.

### I.—FOOD AND FOOD VALUES.

### The Albuminates.

Before entering on the consideration of the question of "Diet in Disease," it is of the first importance that the processes of digestion and assimilation of food in the body should be thoroughly understood, as well as the composition and the exact values of those foods which serve to build up the body after wear and waste, and to maintain it in a condition of health. I will, therefore, commence this series of papers by giving a brief description of the constitution and dietetic values of the various kinds of food which form the mixed diet of an ordinary European, and also some account of the processes of digestion, absorption, and excretion.

*The human body is composed of the following elements:* Carbon, hydrogen, oxygen, nitrogen, sulphur, phosphorus, chlorine, iodine, potassium, calcium, magnesium, and iron. The first four are present in far larger proportion than the rest. In order that the body may be reconstituted and nourished, all these elements must be represented in the food of man.

*Food is composed of organic and inorganic materials.* The organic materials are furnished both by the animal and the vegetable kingdom, and are composed of the following elements: Carbon, hydrogen, oxygen, nitrogen, sulphur, and phosphorus. Of these oxygen is necessary for the oxidation of the tissues, that is to say, their combination with oxygen in the processes of life and function; carbon is necessary for the production of heat, which is caused by the combination of carbon with oxygen to form carbonic acid gas; hydrogen is necessary in order to combine with oxygen and form water; and nitrogen is all important, as it is the essential element in the composition of the living tissues of the nerves, muscles, brain, blood, as well as of the secretions and juices of the body. Its presence is also necessary in all the vital processes, for without it no energy can be produced, nor can any of the changes take place which are characteristic of the living state in the body.

*Foods are divided into Nitrogenous and Non-nitrogenous*, according as they contain the element of nitrogen or not. In the following table the various principles of food are classified and arranged:—

| | | |
|---|---|---|
| Nitrogenous | 1. Albuminates | Albumen<br>Fibrin<br>Caseine<br>Gluten<br>Gelatine |
| Non-nitrogenous | 2. Fats or Hydro-Carbons | Oil<br>Butter<br>Margarine, &c. |
| | 3. Carbo Hydrates | Starch<br>Dextrine<br>Cane-Sugar<br>Grape-Sugar or Glucose<br>Milk-Sugar or Lactose |
| | 4. Stimulants | Alcohol<br>Tea<br>Coffee<br>Cocoa |
| Inorganic | | Water<br>Vegetable acids: Acetic, Tartaric, Citric, and Malic acids.<br>Salts: Chloride of Sodium (common salt), Chloride of Potassium, Carbonate of Calcium (lime), Phosphates of Calcium and Magnesium, &c. |

*The Albuminates* contain about 15 per cent. of nitrogen. The following analysis by Malder clearly explains their composition:—

| | |
|---|---|
| Nitrogen ... ... ... ... ... | 15·5 |
| Carbon ... ... ... ... ... | 53 5 |
| Hydrogen ... ... ... ... ... | 7·0 |
| Oxygen ... ... ... ... ... | 22·0 |
| Sulphur ... ... ... ... ... | 1·6 |
| Phosphorus ... ... ... ... | 0·4 |
| | 100·0 |

*Albumen* is composed of carbon, oxygen, hydrogen, nitrogen, with some sulphur and phosphorus. It exists in its purest form in the white of an egg, and is characterized by being coagulable by heat. Albumen is an important constituent of all flesh foods, and of eggs and milk. It is also present in a great number of vegetable products, and is found in wheat, oats, Indian corn—hence in bread, oatmeal, and Indian meal—in barley, rye, rice, buck-wheat, beans, peas, lentils, bananas, potatoes, almonds and nuts, and in small quantities in carrots, parsnips, turnips, and artichokes. It is present in its most digestible form in the flesh of animals. Some vegetables contain albumen in large quantities, and there is in fact a larger amount of albumen in a pound of peas than in a pound of beef.

*Fibrin* is almost identical with albumen, but it contains more oxygen and sulphur. It is a constituent of the blood, and undergoes spontaneous coagulation out of the body.

*Caseine* is a component of milk, from which it is thrown down by the action of an organic acid, such as rennet. It is caseine which constitutes the curd of milk, the curdling being effected by the production of free lactic acid during the process of the souring of the milk. Caseine is also the basis of cheese, and in this form it is a highly nitrogenous food. Besides the four elements, carbon, oxygen, hydrogen, and nitrogen, caseine also contains sulphur, but no phosphorus, and is remarkable for the large quantity of phosphate of lime which it is capable of holding bound up with it, and the tenacity with which it retains it. (Pavy.)

*Gluten* is the tenacious, sticky material which is left when flour is kneaded with water and afterwards washed to remove the starch. According to the report of the Paris Gelatine Commission, which sat for ten years making continual researches on the value of the albuminates and gelatine as articles of food, it is stated that gluten is alone necessary to support life. This assertion has since been disputed. To gluten, however, bread, the staff of life, owes its high nutritive qualities.

*The Albuminates, or Nitrogenous Foods*, were formerly designated by the older writers on dietetics as flesh-

* Author of "The Micrometric Measurements of the Blood Corpuscles and the Estimation of their Hæmoglobin," "The Third or Invisible Norris Corpuscle," "On the Formation of Fibrine," and translator of "Cornil and Ranvier's Pathological Histology."

formers, the hydro-carbons, or non-nitrogenous foods, being classified as force-producers. Recent researches have, however, shown that this sharp division of foods into nitrogenous, or flesh-formers, and non-nitrogenous, or force-producers, cannot any longer be maintained. It has been proved by experiment that the muscles do not undergo waste during exercise, which waste has to be restored by nitrogenous food, in anything like the degree which was formerly thought, and which was taught, by Liebig. In fact, the amount of tissue waste in muscular exercise is small, and hence the amount of nitrogenous food necessary for repair is also small. The life and health, however, of all the organic nitrogenous tissues, fluids, and secretions can only be maintained by constant change. As the blood circulates through the body, carrying the elements of nutrition to the furthermost limits of the tissues, it modifies all with which it comes in contact; here parting with some element in order to promote cell secretion or nutrition, there taking up products destined either for excretion or for further elaboration. In order that these constant cell changes, this production of secretion and excretion, and these processes of elaboration and assimilation should go on, the presence of nitrogen is necessary. Hence one of the great uses of the albuminates in the food is to promote the changes of nutrition in the body, and this process is called metabolism. Thus we see that albumen is a necessary food, not only in that it repairs tissue waste, but also because it plays a large part in the production of functional activity and energy. It is, moreover, capable it seems of being split up by the agency of the cells in the body into nitrogenous and non-nitrogenous principles; for we find that when an animal has been fed exclusively on albumen both fat and sugar have been produced from this food within the body, and hence it is possible that albumen under certain conditions may also play the part of a force-producer, force being produced, as I will show later, by the combustion of fat and sugar in the body.

To sum up: *The Uses of Albuminates in the body* are threefold, viz.: (1) To repair the waste of those tissues which contain nitrogen, viz., the muscles, nerves, brain, &c., and to reconstitute the secretions and fluids of the body, and the digestive juices; (2) to control, stimulate and support the vital processes of functional activity and nutrition, and to promote oxidation in the body; (3) to contribute to the development of muscular and nervous energy, by splitting up into nitrogenous and non-nitrogenous elements, by the production of heat, and under certain conditions by the formation of fat.

*The Amount of Albuminous Food necessary.*—A certain amount of albuminous food is necessary for the repair of the body, which is wasted, even in those who live sedentary lives, by the constant action and change going on in the organs; but there is but little doubt that the quantity of flesh foods generally consumed by the well-to-do Englishman is far in excess of the amount of nitrogenous material required to repair tissue waste, and to promote secretion. The exact amount of nitrogenous food necessary to barely support life, or to maintain the body in health with a moderate amount of labour or exercise, or on which to do hard labour, such as that of a navvy or engineer, has been accurately determined by experiments carried on on a large scale in armies and prisons. Thus a prisoner sentenced to less than seven days' imprisonment without hard labour is fed on 1 lb. of bread, with two pints of oatmeal gruel made of 2 oz. of oatmeal to the pint. For twenty-one days' imprisonment the bread is increased to 1½ lbs. a day. The amount of nitrogenous material in this diet is only 2½ oz. It is the lowest diet on which life can be maintained with health, but without hard labour. An English soldier on home service receives 1 lb. of bread and ¾ lb. of meat, which represents nearly 4 oz. of nitrogenous material. For hard labour the nitrogenous material should be increased to nearly 6 oz., which would be represented by about 1½ lb. of bread and 1 lb. of meat. From these facts the conclusion will, I think, be easily drawn that the ordinary Englishman and Englishwoman of the middle classes, who perform no hard labour, and live, as a rule, sedentary lives, consume far too much nitrogenous food in the meat, eggs, milk, and fish taken in three square meals a day. In the accounts of centenarians recently collected by Sir George Humphry, it was shown that those who reached advanced old age in good health were those who lived sparingly.

The albumen which is taken with the food undergoes, after digestion, and the assimilation of the elements necessary for tissue construction, retrogressive changes, and is finally thrown into the blood in the form of urea. This urea must be excreted by the kidneys, and therefore if an excess of albuminous food is taken a great burden is thrown upon the kidneys, which may result in producing disease in these organs. In youth, when growth is rapid, a much larger amount of nitrogenous food is required than in old age, when tissue change is slow. Also those engaged in hard labour require a larger amount of nitrogenous food than those who live sedentary lives.

*Gelatine* is derived from bone and fibrous tissues by boiling. It has the property of solidifying into a jelly or cooling. It approaches an albuminate in chemical composition and is rich in nitrogen. It cannot, however, replace albumen in the repair of tissue waste, as it rapidly undergoes change in the body and is eliminated as urea. But just because it undergoes changes so rapidly and easily in the body it can be taken as a substitute for albuminates when stronger foods containing albumen cannot be tolerated. This is the rationale of the use of beef-tea—which is simply gelatine—in cases of acute sickness, when meat cannot be digested. It also contributes to force production.

